# Curcumin Prevents Ototoxicity Induced by Cisplatin as Evaluated with OAE

**DOI:** 10.22038/IJORL.2023.71786.3452

**Published:** 2023-07

**Authors:** Muhammad Dana Arwanda, Tengku-Siti-Hajar Haryuna, Indri Adriztina, Khalisanni Khalid

**Affiliations:** 1 *Department of Otorhinolaryngology - Head and Neck Surgery, Faculty of Medicine, Universitas Sumatera Utara, Medan, Indonesia*; 2 *Malaysian Agricultural Research and Development Institute (MARDI), MARDI Headquarters, Persiaran MARDI-UPM, 43400 Serdang, Selangor, Malaysia.*

**Keywords:** Cisplatin, Curcumin, Cochear, Ototoxicity, Rattus norvegicus

## Abstract

**Introduction:**

This study, assess the efficacy of curcumin therapy in preventing/improving cochlear damage in Rattus norvegicus ototoxic model as evaluated with OAE examination.

**Materials and Methods:**

This study utilized 27 rats which had been injected with single dose 8mg/KgBB of cisplatin, and then divided into 3 groups. The first group was not given curcumin. The second group received curcumin at a dose of 150 mg/KgBB. The third group received curcumin at a dose of 300 mg/KgBB. Curcumin was given from the time of single dose cisplatin injection (day 0) to day 7. OAE examination was performed on the day before the single dose of cisplatin (day 0), day 3, 4 and 7 after curcumin administration.

**Results:**

There was a decline in the average value of SNR in all groups starting from high frequency. However, in the rat groups receiving curcumin there was a slow decrease in the SNR value, which also obtained statistically significant differences in the SNR values of all groups.

**Conclusion:**

Administration of certain doses of curcumin may reduce the modest and statistically insignificant decrease in mean SNR values indicating a reduction in ototoxicity from cisplatin.

## Introduction

Ototoxic

With ototoxicity, there are adverse pharmacological reactions secondary to drugs that can affect the inner ear or auditory nerve, which is characterized by cochlear or vestibular dysfunction (1,2). The prevalence of hearing loss after cisplatin treatment is dependent on the cumulative dose, and has been reported to be as high as 90% (3). Cisplatin is a widely used chemotherapeutic agent with a high rate of ototoxicity having an average incidence of more than 60%. Up to 60% of pediatric patients receiving cisplatin and carboplatin present with an ototoxic event (4).

The basis of this ototoxicity has not been fully elucidated. Generally, it is known that hearing loss is caused by excessive production of reactive oxygen species by cochlear cells. Furthermore, more recent data suggest that inflammation also triggers inner ear cell death through endoplasmic reticulum stress, autophagy and necroptosis, which leads to apoptosis (5). 

Inflammation is one of the first events triggered by cisplatin exposure, leading to a cascade of reactions culminating in oxidative stress and the production of reactive oxygen species (ROS). ROS subsequently induce lipid peroxidation, protein nitration, DNA alteration, amplification of the inflammatory process and even cell death. To restore homeostasis, cochlear cells respond to cisplatin by activating protein and DNA repair mechanisms. Failure of the cellular defense system against ROS-induced damage can lead to cell death (5,6). 

Curcumin, the active ingredient of the plant Curcuma longa, has received great attention over the past two decades as an antioxidant, anti-inflammatory, and anticancer agent (7-10). Early detection of hearing loss caused by ototoxic drugs has become a worldwide research goal. Otoacoustic Emission (OAE) represents the functional status of the outer hair cells and is the only non-invasive means to objectively assess the cochlea. Therefore, this examination is particularly sensitive for monitoring the effects of ototoxicity on cochlear function (11). There are several animal studies conducted using OAE. Likewise, studies using curcumin, is known to have many beneficial effects on the body. It was shown that the antioxidant properties of curcumin are likely to delay and prevent the progression of cochlear damage biomolecularly (12).

A previous study showed that curcumin extract from turmeric (Curcuma longa L.) is capable of preventing noise-exposed rat cochlear damage at molecular biology level (protein level) by decreasing apoptosis index, oxidant status, and increasing antioxidants.^13^ However, to date, there is no specific treatment to prevent cochlear damage caused by cisplatin. In particular, there have been no studies assessing the effect of curcumin in preventing the effects of cisplatin from damaging the cochlea evaluated by the Signal to Noise Ratio (SNR) value with OAE examination. Regarding this, the authors were interested in conducting a study on the effect of curcumin administration as a preventive agent for cisplatin-induced hearing loss assessed by OAE examination in Rattus norvegicus. 

This study is expected to be a motivation and reference for further research into humans and develop into a phytopharmaceutical product that can be used generally in the prevention of cochlear damage due to cisplatin.

## Materials and Methods

This study is an ex vivo experimental laboratoric study with a pre test post test design with control group to determine the effect of curcumin administration on cochlear damage due to cisplatin which is assessed by otoacoustic emission (OAE) examination. Each experimental group was subjected to OAE examination on the 0th, 3rd, 4th and 7th days, after curcumin administration (14,15).

The groups were divided randomly and the control group served as the comparison. This study will be conducted in a standardized laboratory with complete equipment and adequate experience. Maintenance and treatment of experimental animals will be carried out at the Animal House, Faculty of Mathematics and Science, Universitas Sumatera Utara.

***Samp*** ***le***

The samples used in this study were Rattus norvegicus Wistar rats, male sex, healthy condition, adult age (2-3 months), with a body weight of 200-250 grams to ensure relatively small changes in weight during the study. The minimum sample size was 27 rats for three groups, according to Federer's formula calculation. After the sample adapted to the cage environment in the laboratory for 14x24 hours, the treatment was given according to the planned group.

Grouping of research samples was divided into 3 groups. Each group of rats was given a single dose of cisplatin at a dose of 8 mg/kgBB rat. Group 1 served as the control group, which is a group of rats without administration of curcumin. Group 2 is a group of rats given curcumin at a dose of 150 mg / kgBB, while group 3 is a group of rats given curcumin at a dose of 300 mg / kgBB (16). Prior to treatment, a screening was conducted with the inclusion criteria of rats with SNR values ≥ 3 dB based on OAE examination results before treatment (17). Rat were excluded if they were diagnosed with disease by the consultant veterinarian, either infectious or non-infectious diseases or physical injury or potential disease transmission within the period of clinical evaluation (acclimatization) in appropriate environmental conditions (for 14 x 24 hours) (18), Rat with congenital abnormalities, aggressive behavior (often attacking other rat), and rat that died before treatment or examination.

*Procedure*

To ensure that all procedures performed in this study are ethically acceptable, prior to the research the proposal is submitted first to the Research Ethics Commission of the Faculty of Medicine, Universitas Sumatera Utara to obtain an assessment and ratification of ethical eligibility with number: 811/KEP/USU/2021.

The samples were given intraperitoneal injection of cisplatin at a dose of 8 mg/kgBB single dose with a 1 cc 28 G syringe, followed by measurement of the study variables in each treatment group. Each sample in the study group had its SNR value measured with OAE before treatment. The mice were divided into 3 groups. Curcumin was given from the time of cisplatin injection (day 0) until day 7. OAE examination was carried out on the day before cisplatin administration (day 0), day 3, 4 and 7 after cisplatin administration.

Prior to OAE examination, an inspection of the mouse ear canal was carried out and ensured that it was clean and there were no other abnormalities. Afterwards, the mice were anesthetized with Ketamine Hydrocloride at a dose of 50 mg/kgBB which was injected peritoneally (19). 

The OAE examination was performed with the smallest probe (commonly used in infants) with the probe tube inside the rubber probe. OAE assessment was measured at a frequency of 1.5 kHz-6 kHz in the form of Signal to Noice Ratio (SNR) values with normal SNR values ≥ 3 dB (17).The curcumin was given directly to the stomach of rats in forms of solution suspended as much as 2.5 ml using gavage, where curcumin with a dose of 150 mg/ KgBB and 300 mg/ KgBB was first suspended in 0.5% CMC (CMC was made by dissolving 0.5 g CMC in 100 cc of distilled water solution).

Statistical Analysis

The research data obtained will be processed and analyzed univariately, bivariately and multivariately using SPSS (Statistical Package for the Social Sciences). The normality of data was assessed by the Shapiro Wilk test.

## Results

**Fig 1:**
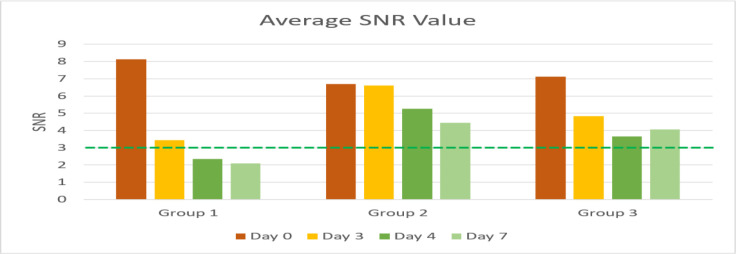
Frequency distribution of average SNR values in each group

**Table 1:** Frequency distribution of average SNR values for each frequency in each group

Frequency	Day	Group		
		1	2	3
		Mean ± SD	Mean ± SD	Mean ± SD
**1500hz**	Day-0	3,33 ± 0,59	3,22 ± 3,12	3,56 ± 3,17
	Day-3	3,11 ± 0,47	3,78 ± 3,12	3,33 ± 3,29
	Day-4	3,17 ± 1,38	4,33 ± 4,16	4,39 ± 4,22
	Day-7	2,67 ± 1,03	2,56 ± 2,66	3,17 ± 3,13
**2000hz**	Day-0	3,72 ± 2,30	3,83 ± 3,11	3,56 ± 3,17
	Day-3	2,72 ± 1,45	2,89 ± 3,68	3,83 ± 3,73
	Day-4	3,33 ± 1,50	2,61 ± 2,83	2,94 ± 3,00
	Day-7	3,28 ± 1,56	1,33 ± 1,81	2,89 ± 2,61
**3000hz**	Day-0	6,44 ± 3,18	4,50 ± 4,59	5,56 ± 4,44
	Day-3	5,28 ± 2,72	5,17 ± 3,07	4,00 ± 3,55
	Day-4	3,50 ± 3,03	3,17 ± 3,67	3,33 ± 2,97
	Day-7	3,06 ± 1,95	3,11 ± 2,93	4,72 ± 3,71
**4000hz**	Day-0	10,72 ± 3,36	7,89 ± 7,90	10,00 ± 7,93
	Day-3	7,78 ± 2,96	7,17 ± 6,49	5,28 ± 3,74
	Day-4	2,78 ± 2,90	8,33 ± 6,02	4,61 ± 3,97
	Day-7	2,44 ± 2,45	7,56 ± 5,55	5,61 ± 4,85
**5000hz**	Day-0	10,72 ± 3,06	9,61 ± 10,78	8,00 ± 9,31
	Day-3	1,39 ± 1,91	9,11 ± 5,48	7,83 ± 6,31
	Day-4	1,11 ± 1,18	7,17 ± 6,34	4,06 ± 5,15
	Day-7	0,89 ± 1,32	7,33 ± 6,44	5,11 ± 5,44
**6000hz**	Day-0	13,78 ± 4,02	11,06 ± 12,36	12,11 ± 11,71
	Day-3	0,39 ± 0,78	11,50 ± 7,20	4,67 ± 6,47
	Day-4	0,22 ± 0,65	6,00 ± 7,05	2,61 ± 3,57
	Day-7	0,22 ± 0,55	4,72 ± 5,81	2,83 ± 4,02

Based on Table 1, the mean SNR value after injection of cisplatin 8 mg/KgBB has decreased on day 3 and intensified on days 4 and 7 in all groups. From Figure 1, the average decrease in SNR occurred in all groups, but in the groups treated with curcumin (groups 2 and 3) there was a decrease in SNR value but only slightly. Although there was a decrease in SNR values in groups 2 and 3, the average SNR value in these groups was still within normal limits.

**Table 2:** Kruskal-Wallis test of mean SNR values between groups on days 3, 4 and 7

Group	Hari							
		Day 3		Day 4			Day 7	
			p		p			p
1	Median IQR Max Min	3,67	0,001	2,17	<0,001	2,08		<0,001
		1,30		1,71		1,42		
		6,00		4,33		3,83		
		1,17		0,83		0,83		
2	Median IQR Max Min	6,17		4,83		4,09		
		4,71		2,88		2,42		
		12,17		12,00		8,17		
		1,83		1,17		2,17		
3	Median IQR Max Min	4,17		3,17		3,25		
		2,09		1,71		3,42		
		11,00		7,67		9,67		
		3,00		1,67		1,33		

*IQR = interquartile range; Max = Maximum; Min = Minimum

From Table 2, it is noted that there is a significant difference in the average SNR value between groups 1, 2 and 3 on days 3 (p=0,001), 4 (p<0,001) and 7 (p<0,001). 

To determine the difference in average SNR values between the two groups, the Mann-Whitney test was performed, which can be seen in Table 3.

**Table 3:** Mann-Whitney test of mean SNR values between groups on days 3, 4 and 7

Group	Day					
	Day 3 Median (IQR)	p	Day 4 Median (IQR)	p	Day 7 Median (IQR)	p
**1** **2**	3,67 (1,30)	0,001	2,17 (1,71)	<0,001	2,08 (1,42)	<0,001
	6,17 (4,71)		4,83 (2,88)		4,09 (2,42)	
**1** **3**	3,67 (1,30)	0,022	2,17 (1,71)	0,006	2,08 (1,42)	0,002
	4,17 (2,09)		3,17 (1,71)		3,25 (3,42)	
**2** **3**	6,17 (4,71)	0,057	4,83 (2,88)	0,009	4,09 (2,42)	0,366
	4,17 (2,09)		3,17 (1,71)		3,25 (3,42)	

*IQR = interquartile range

Based on Table 3, the mean SNR value on day 3 showed a significant difference in SNR value between group 1 and group 2 (p=0,001) and group 1 and group 3 (p=0,022). The mean SNR value on day 4 showed a significant difference in the mean SNR value of each group tested. The mean SNR value on day 7 was found to have a significant difference in SNR value between group 1 and group 2 (p<0,001) and group 1 and group 3 (p=0,001).

**Table 4:** Friedman test of mean SNR values on days 0, 3, 4 and 7 in each group

Day	Group							
		1		2		3	
			p		p		p
Day-0	Median IQR Max Min	8,00 3,21 11,50 4,67	<0,001	5,00 3,67 19,33 2,67	0,096	5,17 2,88 20,17 1,83	0,007
Day-3	Median IQR Max Min	3,67 1,30 6,00 1,17		6,17 4,71 12,17 1,83		4,17 2,09 11,00 3,00	
Day-4	Median IQR Max Min	2,17 1,71 4,33 0,83		4,83 2,88 12,00 1,17		3,17 1,71 7,67 1,67	
Day-7	Median IQR Max Min	2,08 1,42 3,83 0,83		4,09 2,42 8,17 2,17		3,25 3,42 9,67 1,33	

*IQR = interquartile range; Max = Maximum; Min = Minimum

According to Table 4, there is a significant difference in the average SNR value in group 1 on days 0, 3, 4 and 7 (p<0,001). In group 2 there was no significant difference in the average SNR value on days 0, 3, 4 and 7 (p=0,096). In group 3, there was a significant difference in the average SNR value on days 0, 3, 4 and 7 (p=0,007). 

According to Table 5, the Wilcoxon test was carried out to determine the statistical difference in the average SNR values in each group between days of examination.

**Table 5:** Wilcoxon test of mean frequency on days 0, 3, 4 and 7 in each group

Day	Group					
	1		2		3	
	Median (IQR)	p	Median (IQR)	p	Median (IQR)	p
**Day-0** **Day-3**	8,00 (3,21)	<0,001	5,00 (3,67)	1,000	5,17 (2,88)	0,018
	3,67 (1,30)		6,17 (4,71)		4,17 (2,09)	
**Day-0** **Day-4**	8,00 (3,21)	<0,001	5,00 (3,67)	0,408	5,17 (2,88)	0,010
	2,17 (1,71)		4,83 (2,88)		3,17 (1,71)	
**Day-0** **Day-7**	8,00 (3,21)	<0,001	5,00 (3,67)	0,240	5,17 (2,88)	0,031
	2,08 (1,42)		4,09 (2,42)		3,25 (3,42)	
**Day-3** **Day-4**	3,67 (1,30)	0,006	6,17 (4,71)	0,177	4,17 (2,09)	0,064
	2,17 (1,71)		4,83 (2,88)		3,17 (1,71)	
**Day-3** **Day-7**	3,67 (1,30)	0,001	6,17 (4,71)	0,220	4,17 (2,09)	0,218
	2,08 (1,42)		4,09 (2,42)		3,25 (3,42)	
**Day-4** **Day-7**	2,17 (1,71)	0,447	4,83 (2,88)	0,155	3,17 (1,71)	0,446
	2,08 (1,42)		4,09 (2,42)		3,25 (3,42)	

*IQR = interquartile range

In Table 5, it was found that in group 1 there was no difference in the average SNR value only on day 4 and day 7 (p=0,447). In group 2 there was no difference in the average SNR value between examinations, while in group 3 there was no difference in the average SNR value on day 3 and day 4 (p=0,064), day 3 and day 7 (p=0,218), and day 4 and day 7 (p=0,446).

## Discussion

Platinum-based chemotherapy has been widely used and is particularly effective against solid tumors involving the head and neck, lungs, ovaries, testis, and bladder in adults. In children, platinum compounds are commonly used in the treatment of neuroblastoma, osteosarcoma, hepatoblastoma, germ cell and central nervous system tumors. 

The three main platinum compounds (cisplatin, carboplatin, and oxaliplatin) differ in terms of their chemical structure and side effect profile. The main adverse effect associated with cisplatin is irreversible sensorineural hearing loss (20).

Chemotherapy (antineoplastic) agents, especially those containing platinum (cisplatin and carboplatin), can lead to tinnitus and hearing loss. Hearing loss can be both severe and permanent, occurring immediately after the first dose, or can be delayed until several months after completion of treatment (21).

This leads to an initial increase in hearing threshold at high frequencies, followed by progressive loss to lower frequencies if therapy is continued (22). 

Cisplatin mediates the activation of Nicotinamide Adenine Dinucleotide Phosphate Oxidase 3 Isoform (NOX3) and regenerates Reactive Oxygen Species (ROS). The generation of ROS promotes Signal Tranducer and Activator of Transcription 1 (STAT1) to be activated which will stimulate the inflammatory process. The association of activated STAT1 with activated p53 promotes apoptosis in cochlear cells (23).

In a previous study conducted by De Freitas et.al, a significant decrease was observed in SNR values at frequencies of 3,000-8,000Hz between days 0 and 3 in a group of rats receiving cisplatin. Day 4 resulted in the death of the rats and one rat had no SNR value at 6,000Hz (14). In the study conducted by Somdas et.al, there was a decrease in DPOAE values and ABR thresholds in the rat group after receiving cisplatin between days 0 and 7 (15).

Therefore, we conducted the study on days 0, 3, 4 and 7 after cisplatin administration. In addition, as seen from the results, the decrease in SNR values was found to decrease on the third day onwards after the administration of 8 mg/kgBB cisplatin in each group starting from a frequency of 3,000Hz.

Curcumin (1,7-bis (4-hydroxy -3-methoxyphenyl) -1,6-heptadiene-3,5-dione), also termed as diferuloylmethane, is the main natural polyphenol found in Curcuma longa (turmeric) and other Curcuma spp.^7^ Curcumin, a form of polyphenol, has been shown to act on several signaling molecules, while also showing a multitude of activities at cellular level, which has aided in supporting healing with its many health benefits. It has been shown to benefit from various inflammatory conditions and metabolic syndromes (24,25).

From the graph shown in Figure 1, it was found that the average SNR value decreased in each group, except for the group given curcumin (groups 2 and 3), the average SNR value was still within normal limits.

According to Table 2, there is a difference in the average SNR value between groups on days 3, 4 and 7. As shown in Table 3, there are differences in the SNR values of the groups that were not given curcumin and the groups that were given curcumin. It shows that the average SNR value in the groups given curcumin (groups 2 and 3) is still within normal limits, as evidenced by statistically significant differences between the groups that did not receive curcumin and those that received curcumin on days 3, 4, and 7.

Previous studies have shown that polyphenols administered together with cisplatin can exert anti-oxidant and pro-oxidant effects. However, polyphenolic compounds exhibit different mechanisms of action depending on the cell context and dosage (8). 

According to the results of the study, there was no difference in the mean SNR values in groups 2 and 3 on days 3 and 7, but there was a difference in the mean SNR values on day 4 in Table 3. This shows that different doses affect the ototoxicity effect of cisplatin.

The bioavailability of curcumin and its antioxidant ability were not influenced by increasing the dose of curcumin. In addition, curcumin is more effective in enhancing antioxidant capacity when consumed in small amounts. Consequently, curcumin is a nutritional hormone that follows the hormonal pathway. The response to hormonal doses is biphasic and characterized by a dose-response relationship divided into two dose zones, namely low doses with stimulatory responses and high doses with inhibitory reactions and side effects. Preliminary experimental research in previous studies showed concentration-dependent anti- and prooxidant properties of curcumin (26).

In the study of Paciello et.al, the use of curcumin at a dose of 200 mg/KgBB was able to reduce the ototoxicity of cisplatin, but low doses (100 mg/KgBB) did not have an effect and high doses of 400 mg/KgBB even worsened it. In line with table 4, there was a significant difference in mean SNR values in groups 1 and 3 between days 0, 3, 4 and 7, not in group 2, supported by table 5 where there was no significant difference in mean SNR values in group 2, indicating that optimal doses can reduce the ototoxicity of cisplatin, while increased doses do not affect the ototoxicity of cisplatin.

In accordance with the results of previous studies, it was shown that selected polyphenols can lower the hearing threshold, and simultaneously, these compounds interact with the signaling pathway of ROS, inducing an adaptive stress response by regulating the translocation of Nrf-2. Furthermore, it has been observed that the expression of NF-kB activated by cisplatin is upregulated only by curcumin in the primary cochlear structures (27), here illustrating the anti-inflammatory effect of curcumin. Also, both the antioxidant and anti-inflammatory effects of polyphenols cause inhibition of phosphorylation of p53 thereby decreasing the death of outer cochlear hair cells, i.e. the main population of cochlear hair cells affected by the drug cisplatin.^8^

## Conclusion

Despite the decrease in SNR values in OAE examinations in all groups, at certain doses curcumin can slow down cochlear damage, which is evident from the difference in SNR reduction values that are statistically significant against the group of rats not given curcumin, both with the group of rats given curcumin 150 mg/KgBB and the group of rats given curcumin 300 mg/KgBB. At certain doses curcumin can reduce the ototoxicity of cisplatin. We expect that curcumin can continue to be studied with better research designs or methods in the future.
